# Are People With Type 1 Diabetes Mellitus Appropriately Following Insulin Injection Technique Practices: A Review of Literature

**DOI:** 10.7759/cureus.51494

**Published:** 2024-01-01

**Authors:** Swar Gupta, Harshal Ramteke, Shlok Gupta, Sunil Gupta, Kavita S Gupta

**Affiliations:** 1 Medicine and Surgery, Jawaharlal Nehru Medical College, Datta Meghe Institute of Higher Education and Research, Wardha, IND; 2 Surgery, Jawaharlal Nehru Medical College, Datta Meghe Institute of Higher Education and Research, Wardha, IND; 3 Internal Medicine, Sunil's Diabetes Care n' Research Centre, Nagpur, IND; 4 Diabetology, Sunil's Diabetes Care n' Research Centre, Nagpur, IND; 5 Nutrition, Sunil's Diabetes Care n' Research Centre, Nagpur, IND

**Keywords:** recurrent hypoglycemia, medical education, insulin injection techniques, insulin lipodystrophy, type 1 diabetes mellitus (t1dm)

## Abstract

People with type 1 diabetes mellitus (T1DM) need to take multiple doses of insulin injections daily throughout their lives. However, a notable portion of people with diabetes mellitus (DM) show suboptimal insulin injection technique practices. They are supposed to follow the recommended insulin injection technique guidelines. Our explorative literature search, including studies from the past 30 years, is expected to identify the deficiencies of self-injection insulin techniques and the associated complications in people with T1DM, where we have summarised the overall incidence of complications that have occurred due to nonadherence of the prescribed guidelines, along with their associated risk factors. We have attempted to include multiple systematic reviews, meta-analyses, literature reviews, case reports, and original articles from the search engines and databases like PubMed, Scopus, ScienceDirect, Cochrane Library, Google Scholar, and BioMed Central, and studies with only human participants were included in this search. The knowledge sharing from this research may be utilised for enhancing the structured education diabetes programme and implementing the population-based corrective measures, including the thrust areas in future multi-centre longitudinal research studies and recommendations, which can prevent unnecessary complications and enhance their quality of life. Correct insulin administration technique, abstaining from administration of injection at the areas with lipohypertrophy, rotation of injection sites, and ultrasound scanning can be used as a complimentary method to detect the lipohypertrophy at an early stage. Liposuction is beneficial in reducing the extensive lipohypertrophic tissues but helps achieve only cosmetically satisfactory outcome; thus, empowering people to follow insulin injection technique guidelines is one of the best strategies to reduce the high prevalence of lipohypertrophy. To conclude, education among the people with DM, especially T1DM who have to take insulin regularly, needs to be carried out consistently in the clinical settings, to prevent the severe complications caused due to inappropriate insulin injection techniques.

## Introduction and background

Diabetes mellitus (DM) encompasses a group of common and prevalent disorders related to metabolism that share the phenotype of hyperglycemia [1]. Type 1 diabetes mellitus (T1DM) occurs due to autoimmunity against the beta cells of the pancreas that are involved in the formation of insulin, which results in insulin deficiency, whereas impaired insulin secretion, varying degrees of insulin resistance, and enhanced hepatic glucose production are the salient features of type 2 diabetes mellitus (T2DM) [1]. Insulin has served as the primary therapeutic approach for individuals with T1DM in the last century, aiding them to attain their desired glycemic targets [2]. Insulin is available in rapid-, short-, intermediate-, and long-acting types, administered individually or combined in a single syringe for injection [3]. Insulin is typically delivered subcutaneously, using insulin pumps or various devices for multiple daily injections [4]. Exogenous insulin must be delivered into the subcutaneous tissue rather than through the intramuscular (IM) route. The preferred sites for administering insulin encompass the upper arm, abdomen, buttock, and thigh. The absorption of insulin from IM sites is also influenced by muscle activity. Unintentional IM injection consequently results in improper insulin absorption and is associated with an increased likelihood of frequent and unexplained events of hypoglycemia [5]. Every person on insulin therapy should follow the recommended insulin administration technique to achieve the desired outcomes. To get the desired results, everyone using insulin therapy should adhere to the suggested insulin delivery technique. Numerous studies have demonstrated that optimising insulin injection procedures yields the highest effect from the treatment [6,7]. Despite having insulin technique guidelines (Forum for Injection Technique (FIT), Forum for Insulin Injection Technique (FIIT), American Diabetes Association (ADA)) in the public domain, many surveys have revealed that a substantial number of individuals with T1DM are not adhering to these recommendations properly [8].

Effective glycemic control hinges on various aspects of insulin injection practices, including insulin storage, potential mixing when using syringes, choosing the right needle length, selecting and rotating injection sites, changing needles as needed, examining injection sites for signs of lipohypertrophy (LH), regular ultrasound scanning for the early diagnosis of suspected LH, and appropriately disposing of used needles [6]. LH is characterised by the formation of firm, elevated lumps in the adipose tissue underneath the skin, resulting from the repeatedly injecting the insulin at a similar site [8]. This condition is closely linked to the insufficient rotation of injection sites for the delivery of insulin, repeated use of needles, duration of DM, etc. Health education programmes and the efficient use of multimedia can play a major role in creating awareness among the masses, thereby enhancing the efficacy of treatment [9]. The people who were actively involved in rotating their injection sites had a reduced occurrence of LH [10-15].

This literature review aims to establish the importance of adhering to the proper insulin injection technique practices. Our objective is to assist healthcare professionals in recognising the high prevalence of LH and its consequences. This article should empower the medical professionals and educators with evidence-based knowledge towards applying the preventive strategies through focused education, especially for those with T1DM.

The research gap that needs to be addressed is that despite knowing that there is a high prevalence of LH in individuals with T1DM, which has its impact on glycemic control, still the awareness of the condition among the T1DMs remains low and the implementation of preventive measures seems to be slow, especially in developing countries like India. Currently, exploratory studies on whether the people with T1DM are taking insulin appropriately, which type of injection technique (e.g. site rotation, reuse of needles, administering injection at multiple sites) needs attention, and what the thrust areas of education are need to be carried out.

## Review

Methodology

A literature search was conducted to comprehensively review the various articles and case reports highlighting the importance of the proper insulin injection technique practices being carried out by the people with T1DM globally across the different regions. This article encompasses the review of multiple original articles, review articles, and case reports from the research databases and search engines such as PubMed, Scopus, ScienceDirect, EMBASE, Cochrane Library, Google Scholar, and BioMed Central. This search was focused from the period of 2010 to 2023, although we had access to the medical literature dating back to 1993 (30-year period). Along with the electronic database searches, lists of references of the numerous relatable review papers and articles were manually selected to identify the relevant studies.

**Figure 1:**
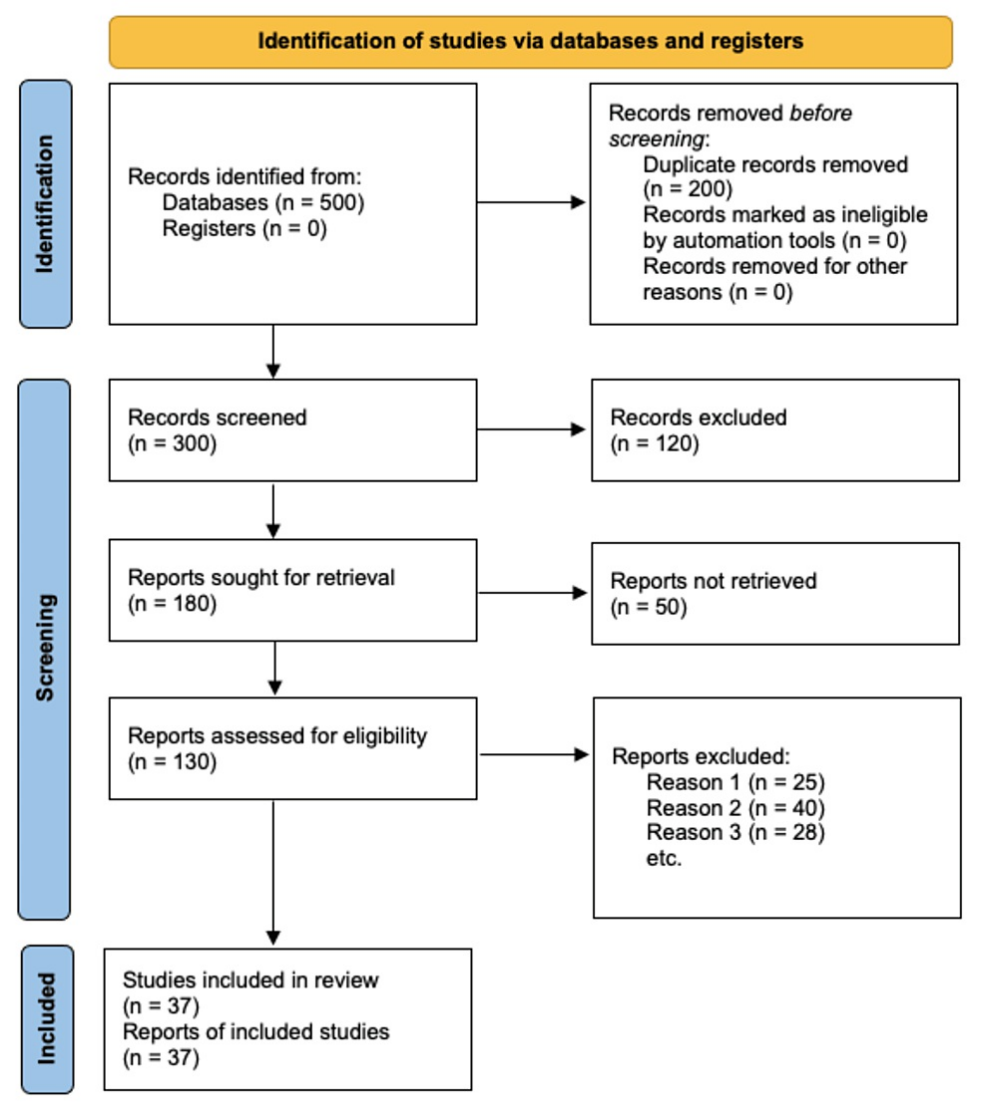
A PRISMA flow diagram employed as a research methodology for the article selection process of the literature review PRISMA: Preferred Reporting Items for Systematic Reviews and Meta-Analyses

The inclusion criteria of this literature review included experimental studies, observational studies, meta-analyses, and systematic reviews that enlisted various outcomes of the interventions involved in screening the insulin injection technique practices among different age groups having T1DM. Only studies with human participants were included while focusing solely on the insulin injection technique practices and complications associated with it. Only peer-reviewed articles were included in the article, while the other sources were excluded. The authors evaluated the full-text articles for eligibility, resolving any discrepancies through discussions and consensus. The comprehensive literature search was conducted with the goal of ensuring the incorporation of pertinent studies and providing a thorough analysis of the insulin injection techniques being followed by people with T1DM. Additionally, we also consulted the experts in this specific field with the sole purpose of identifying the potential issues that need to be addressed and resolved through a combined effort by healthcare professionals like nurses, diabetes educators, and doctors, thereby substantially reducing the complications among people with T1DM. Figure 1 illustrates the selection process of the varied studies utilised for our literature review.

Insulin and its administration in T1DM

Insulin plays a vital role in metabolising normal carbohydrates, fats, and proteins. Individuals with T1DM lack the capability to naturally form sufficient levels of this hormone required for their survival, necessitating their reliance on exogenous insulin [16]. DM is a worldwide epidemic, and the projected figures indicate that the global diabetes population is expected to rise to 643 million by 2030 and further to 783 million by 2045, as claimed by the International Diabetes Federation (IDF). Moreover, India is believed to have approximately 97,700 children diagnosed with T1DM [17]. One of the most common aetiologies of recurrent hypoglycemia and glycemic variability is lipodystrophy, which occurs due to improper insulin injection techniques practices, leading to severe complications. 

Key Recommendations

According to the latest guidelines from the ADA, the children at the school must be assisted and supported in utilising the latest technology, inclusive of continuous glucose monitors (CGMs), automated insulin delivery systems, insulin pumps, and connected insulin pens, as recommended by their diabetes care team [18]. For young individuals managing DM by using multiple injections throughout the day or insulin pump therapy, there must be a choice to utilise either the devices which help in monitoring the real-time CGMs or intermittently scanned CGMs as an available option [18]. HbA1C targets should be individualised for each person and periodically reviewed and adjusted as needed. An HbA1C level below 7% (equivalent to 53 mmol/mol glucose) is considered suitable for most adolescents and children [18].

In accordance with the recent recommendations by the Mayo Clinic, the 4 mm insulin pen and 6 mm syringe needles are prescribed for usage, as they are regarded safe and efficacious, cause less discomfort, and hence should be the primary choice of treatment in all patient categories. It is advised to refrain from IM injections, especially with long-acting insulins, due to their potential risk of causing severe hypoglycemia [2]. LH, a common therapy complication that affects insulin absorption, necessitates injections and infusions to be delivered away from the insulin injection sites, and its appropriate rotation can help prevent them [2]. To ensure the long-term success of insulin therapy, we must also involve addressing psychological barriers such as fear of pain, anxiety due to injections, administering the injection above the clothes in public spaces, and lack of confidence early in the treatment process, even before starting insulin [2]. Improper disposal of used sharp objects can pose a potential risk of blood-borne infections, but the risk can be substantially reduced by the adoption of the following practices such as appropriate training, efficient methods of disposal, and the regular usage of safety devices [2]. Adhering to these updated guidelines will give rise to increased effectiveness of the medical therapies, enhanced outcome, and reduced expenses incurred by individuals with DM [2]. The lack of adherence results in the insufficient management of blood sugar levels, heightened risks of unexplained hypoglycemia, ketoacidosis, glycemic variability, increased hospitalisation rates, elevated therapy costs, and, ultimately, a negative impact on their quality of life [6].

Prevalence of LH in DM

In a study of 119 children who were examined in a teaching hospital in Alexandria, the occurrence of LH was notably linked to the length of time an individual has had DM and their body mass index (BMI). To attain adequate control, children with LH required a noticeably greater dosage of insulin units per kilogram of body weight in comparison with the children who did not have LH [3]. Research in Quebec, Canada, has illustrated that many adult T1DMs using subcutaneous insulin infusion encountered many technical and infusion site-related adverse events (including 45% with LH) [19]. Even in China, the prevalence varies from 53.1% to 73.4% [20]. In another study by the ADA, which included 50.8% of people with T1DM, 80.8% were diagnosed with LH by ultrasound scan, where the three characteristic features were notable thickening of the subcutaneous fat layer, elevated echogenicity in the diffuse areas, and the presence of nodular hyperechoic foci [21]. Amyloid formation related to insulin has been detected across all methods of delivery of insulin, including injection, infusion, and inhalation, and there has been an increase in the number of documented cases considerably since the year 2002 [22]. An additional survey of 16 studies showed a significant variation in the occurrence of LH, ranging from 17% to 75%. Also, the combined prevalence of LH in adolescents and children was 45.16%, whereas in adults, the combined prevalence was 41.3%. Interestingly, the incidence of LH in children seemed to be greater in comparison with the adults, and the overall prevalence of LH in Jordan was 37.3% [23,24].

Aetiology and Risk Factors

One of the major causes that is responsible for insulin-induced LH is the lack of education among the patients. Education plays a major role in making the children, along with their parents, aware of the proper techniques to achieve the desired glycemic targets and prevent the dreaded complications associated with T1DM, such as severe hypoglycemia, diabetic ketoacidosis (DKA), and various systemic disorders. In Africa, a significant majority of participants, specifically over three-quarters (79.5%), were using insulin pens or cartridges beyond their expiry date, 70.5% were not practicing proper rotation of injection sites, and 63% were noted to engage in massage of the skin around the site after administering the insulin [25]. Higher dosages of daily insulin, lack of injection site rotation, and keeping the insulin that is currently being used in the refrigerator were independent correlates of the elevated levels of HbA1C [12]. Additionally, while traveling, if the insulin is left in the locked car with closed windows or if the vial is placed in the baggage, there is a risk of exposure to extremely variable temperatures. Many individuals with T1DM frequently opt for injection through their clothing, particularly when they are time-constrained or find themselves in a public setting [9]. Reusing needles for multiple injections can lead to needle blunting and deformities, potentially resulting in inaccurate dosages being administered during injections. A proper injection site is extremely pivotal for the efficient absorption of insulin through advanced insulin delivery systems.

Adverse Effects and Complications of Improper Insulin Injection Techniques

Inappropriate usage of insulin injections may lead to various complications. Transient and severe unexplained hypoglycemia/hyperglycemia is one of the most common complications of LH, which increases glycemic variability and the risk of DKA. This, in turn, makes it difficult to achieve the desired glycemic targets and the increased need for higher insulin dosage. LH can be associated with pain, bleeding, and bruising at the injection site, especially due to the reuse of needles and poor rotation. High glycemic variability also leads to a higher risk of multiple systemic disorders such as hypertension, cardiovascular diseases, and microvascular complications like nephropathy, retinopathy, and neuropathy in people with DM.

Therapeutic Interventions

There are various aspects by which the healthcare system can intervene at multiple stages during the treatment of people with T1DM in order to minimise the complications associated with improper insulin injection techniques and improve the efficiency of the treatment. The nurses, diabetes educators, and health counselors, along with the doctors and specialists, must counsel and educate the patients regarding the prescribed guidelines by the global and regional organisations for proper administration, storage, and usage of insulin injections. They must prioritise their counseling on the most common issues faced by the people, such as minimising the reuse of needles, carrying out proper injection site rotation, refraining from massaging the skin after injection, and using the appropriate size of the needle according to their age (4 mm is considered safe for children). Moreover, with children with T1DM, the healthcare professionals must educate their caretakers about the significance of prescribed guidelines appropriately. They must also be made aware of the potential complications associated with non-compliance with these recommendations and guidelines.

If people with T1DM are willing to make the choice of CGMs, they must be encouraged to utilise them to monitor glucose levels multiple times daily as these devices can efficaciously detect any undesirable change in the glycemic levels on time, leading to timely interventions in the treatment protocol. All children with T1DM must receive assistance at school for the utilisation of the latest technology, which may include advanced devices such as CGMs, automated insulin delivery systems, insulin pumps, and connected insulin pens, as prescribed by their healthcare team [18]. Multiple studies have shown that just by an intervention of educating the people rigorously and making them aware of the proper site of administration, site rotation, and dos and don'ts of insulin administration techniques, they have been able to achieve their glycemic targets effectively [26-32].

The physical examination had a noticeably high rate of missed diagnosis of LH, especially in people with DM who had received insulin for a brief duration and those with an elevated BMI [21]. Thus, the administration sites for insulin injection must be inspected by healthcare professionals at every clinic visit. Additionally, ultrasound scan must be employed as a complementary technique to effectively and promptly diagnose LH along with characterising the lipohypertrophic areas, which consequently might be helpful in improving glycemic control [21,33,34]. 

Another effective surgical intervention is liposuction, which is a therapeutic method for removing extensive insulin-induced lipohypertrophic tissue and can rapidly yield a cosmetically satisfactory result [35-37]. It may also be beneficial for people who do not respond to the conventional therapies. However, although a large number of researches have proven that liposuction can be performed as a medical intervention, it can assist in only providing relief from the symptoms. Hence ultimately, it is the proper insulin administration technique practices that need to be followed by people with T1DM.

The strength of this literature review is to empower the healthcare professionals to incorporate the practices of continuous education in their clinical setting, especially more pertaining to the children and adolescents with T1DM. The limitation is that we have incorporated most of the search engines except MedlinePlus and Web of Science. Table 1 summarises the entire list of the references selected for the study, and the prominent findings observed in each one of them, along with the summarised assessment of the aspects where the people with T1DM and T2DM fail to adhere to the guidelines, along with overall complications of the same.

**Table 1:** Summary table of the list of references cited in this literature review along with their analysed salient features, like associated risk factors and overall incidence of complications DM: diabetes mellitus; LH: lipohypertrophy; BMI: body mass index; T1DM: type 1 diabetes mellitus; FIT: Forum for Injection Technique; ADA: American Diabetes Association

Sr. no.	Authors	Year	Origin of the study	Associated risk factors present/lack of adherence to the guidelines	Overall incidence of complications and summarised result due to lack of adherence to the guidelines	Summary
1	Powers et al. [1]	2018	Harrison's Principles of Internal Medicine, McGraw-Hill	N/A	N/A	Standard definitions and pathophysiology of DM
2	Frid et al. [2]	2016	Mayo Clinic, USA	N/A	N/A	Latest recommendations for the administration of insulin
3	Omar et al. [3]	2011	Alexandria	48.7% occurrence of LH due to using needle length of 8 mm, duration of DM, and BMI	LH occurred in 54.9% of the patients	Examination of the children and adolescents having LH in T1DM
4	Chaudhury et al. [4]	2017	USA	N/A	N/A	Reviewing the antidiabetic drugs and the complications associated with DM
5	Strauss et al. [5]	2002	Europe	Not checking the injection sites properly, failing to rotate sites, 22% people disposing needles without recapping, and long duration of DM	30% people have LH in a population with 58% T1DMs	Informative study of insulin injection technique practices
6	Dagdelen et al. [6]	2018	Turkey	Usage of 8 mm needles, needle reuse, disposal of sharps improperly, incorrect utilisation of skin fold, and focus on the dwell times under the skin	Higher BMI; higher mean total daily dosage of insulin; mean HbA1c was also increased (9.1%)	Survey of Turkish patients with DM using insulin injection
7	Nakatani et al. [7]	2013	Japan	68 out of 87 patients demonstrated poor to moderate understanding about correct insulin injection techniques	Deranged HbA1c ranging from 7.46±0.09%	Improvement in glycemic levels by re-educating the people about their insulin delivery
8	Tandon et al. [8]	2015	India	N/A	N/A	Understanding the FIT, India, recommendations issued for the proper delivery of insulin
9	Huang et al. [9]	2017	Wiley Online Library	N/A	N/A	Effective education through various media in patients with DM
10	Chen et al. [10]	2021	China	Consistently administering insulin into the sites of LH	Palpable LH, elevated HbA1c	Learning to not inject into areas of LH
11	Kamrul-Hasan et al. [11]	2020	Bangladesh	The abdomen was most common site of injection, not cleaning the injection site before administration, less dwell time, skipping insulin injections, not recapping the used syringes	Painful injections, bruising and bleeding, insulin leakage, higher HbA1c levels, high prevalence of hypoglycemia and hyperglycemia	Complications observed among the people with T1DM in Bangladesh
12	Pozzuoli et al. [12]	2018	Italy	Not spacing injections, not rotating the insulin sites, and keeping insulin in use in the refrigerator	LH seen in 42.9% of the patients, severe hypoglycemia and higher daily insulin doses	The risk associated with LH
13	Yuan et al. [13]	2018	China	Incorrect rotation of sites, incorrect needle reuse, incorrect resuspension procedure of the insulin	High HbA1C of a mean of 8.1±1.5%, high glycemic variability, unexplained hypoglycemia	Relation between technique practices for insulin delivery and variability due to glycemic levels
14	Alhazmi et al. [14]	2020	Saudi Arabia	Reuse of needles, improper needle rotation, inappropriate spacing between insulin doses	19.5% of patients had acidosis, seizures, and hospital admissions; uncontrolled blood glucose levels; bleeding and bruising from injection sites in 58.7% of patients; and 35.3% of patients have LH	Assessing the methods followed for administering insulin injection in the Makkah region
15	Gupta et al. [15]	2018	India	Improper injection site rotation and reuse of insulin syringes	Overall prevalence is 69.8%; high glycemic variability and unexplained hypoglycemia	Clinical implications of LH due to improper techniques in people with T1DM
16	ADA [16]	2003	USA	N/A	N/A	Protocol for administering insulin
17	Kumar [17]	2015	India	N/A	N/A	Incidence among the children with T1DM in India
18	ElSayed et al. [18]	2023	ADA, USA	N/A	N/A	Standards of care in DM for children and adolescents
19	Taleb et al. [19]	2018	Quebec, Canada	N/A	Pain, adhesion, lipodystrophy, and irritation at the insertion site during the usage of subcutaneous insulin infusion	Experiences related to using continuous insulin injection therapy in adults with T1DM
20	Xu et al. [20]	2020	China	Needle reuse, improper needle disposal, high BMI, length of the needle, failure to rotate injection sites	Prevalence of LH ranges from 53.1% to 73.4% in China, unexplained hypoglycemia, large glycemic excursion, poor glycemic control	Prevalence, clinical consequence, and pathogenesis of LH
21	Xu et al. [21]	2020	ADA, USA	39% false-negative rate by physical examination of LH. Hence, ultrasound screening can be utilised as a complimentary method	LH	Missing diagnosis of LH during physical examination and the importance of ultrasound scanning
22	Nilsson [22]	2016	USA	N/A	Insulin amyloid formation	Formation of amyloid at sites of injection
23	Soliman et al. [23]	2022	Qatar and Egypt	Longer duration of DM. Most critical risk factor, reuse of insulin syringe >5 times, failure to rotate injection sites, low level of patient education, location of injection	LH	Factors that affect the LH induced by insulin in children
24	Al Ajlouni et al. [24]	2015	Riyadh and Jordan	Duration of DM, needle size, failure to rotate sites systematically, duration of insulin therapy	Significant LH including grades 1, 2, and 3	LH and risk factors in insulin-treated patients
25	Tosun et al. [25]	2019	Africa	79.5% people with DM using the insulin pen after expiry date; 70.5% individuals not rotating sites; 63% people with DM were massaging the injection site and unaware about proper length and appropriate injection sites; repeated use of needle	High HbA1c (10.48±1.78), higher incidence of LH, bleeding and bruising of the injection sites, high glycemic variability, unexplained hypoglycemia, painful injections, complication frequency was more in patients massaging after insulin injection	Improper rotation of insulin injection sites and incorrect site of administration
26	Huang et al. [26]	2023	California	Continuously delivering high doses of insulin at the same location, repeated trauma to skin	Glycemic variability, hypo- as well as hyperglycemia	Re-education must be made necessary to improve the glycemic outcomes
27	Sahasrabudhe et al. [27]	2016	India	Repeated use of the same location, improper site rotation, incorrect injection technique	Bilateral grade 3 LH of the thighs, unexplained hypoglycemia (6-8 times a month), wide glycemic excursions with high postprandial levels along with episodes of hypoglycemia	Hyperglycemia caused due to LH at the injection site
28	Ucieklak et al. [28]	2022	Krakow, Poland	Localising it to a single site, mainly the abdomen, higher daily dose of insulin, higher BMI	LH in 94.9% of the people with DM, redness and scars in areas of earlier cannula implantation	Treating LH in people with T1DM with an insulin pump
29	Campinos et al. [29]	2017	France	Longer needle length (>4 mm), high BMI, reuse of the needles	Palpable LH, high glycemic variability, unexplained hypoglycemia	Efficient intervention for LH due to insulin in France
30	Smith et al. [30]	2017	UK	Poor rotation of sites of injection, inappropriate needle length	LH, high daily doses of insulin, higher mean HbA1c	Study on LH intervention for creating awareness among people with DM in the UK
31	Gentile et al. [31]	2020	Italy	Reusing needles, neglecting to rotate injection sites, and ice-cold insulin injections	High HbA1c values, increased variations of the blood glucose levels, unexplained hypoglycemic events significantly associated with LH	Survey in a particular region on insulin-induced skin LH in Italy
32	Gentile et al. [32]	2021	Italy	N/A	Extensive variability of blood glucose levels, sudden hypoglycemia	LH in patients with T1DM treated with an insulin pump
33	Bertuzzi et al. [33]	2017	Italy	N/A	LH, high HbA1c, poor glycemic control	Characterising LH by ultrasound screening
34	Hashem et al. [34]	2021	King's College London, UK	Poor rotation of injection sites and predominantly seen concentrated over the abdomen and lower thighs	Grade 1, 2, and 3 LH observed	Morphology of the lipohypertrophic areas studied by ultrasound scan
35	Gandolfi and Thione [35]	2009	Italy	N/A	LH	A case report showing that LH can be treated by liposuction
36	Hardy et al. [36]	1993	ADA, USA	N/A	LH	Severe insulin-induced LH can be treated by liposuction
37	Mangan et al. [37]	2023	USA	N/A	Poor glycemic control, LH most common over the thighs	A systematic review of case reports showing liposuction as an effective intervention to treat LH

## Conclusions

It is extremely necessary to train and educate the people who start insulin injection therapy on various aspects, like proper techniques and steps to be followed during the delivery of insulin on repeated follow-up visits. It is the primary duty of healthcare professionals to prepare individuals with T1DM and T2DM on insulin therapy with all the necessary information, expertise, and proficiency. According to various studies worldwide, people with DM who have displayed poor insulin injection skills have a higher incidence of complications at the injection sites. Surgical interventions such as liposuction can be used to treat severe LH and alleviate the symptoms. Ultrasound screening must be used as a complementary technique for the diagnosis of LH. Enhanced education on proper insulin injection technique practices, with a focus on minimising the reuse of needles, ensuring the proper injection site rotation, and refraining from massaging the skin over the site after administering the injection, should be prioritised consistently, and its importance must be emphasised rigorously.
